# Supplementary material for: Quantitative Proteomics and Molecular Mechanisms of Non-Hodgkin Lymphoma Mice Treated with Incomptine A, Part II
Source: Pharmaceuticals (Basel). 2025 Feb 11;18(2):242. doi: 10.3390/ph18020242 (PMC11858899; doi:10.3390/ph18020242)
Supplement: Supplementary file 1 [file pharmaceuticals-18-00242-s001.zip › Figures and tables Footers.pdf]

Figure S1. Network visualization from the 2,717 proteins in a computational representation through Gene Ontology with enrichment analysis of Biological Process subontology. It shows identified categories, and node size in range 50-200 interactions.

Figure S2. Network visualization from the 2,717 proteins in a computational representation through Gene Ontology with enrichment analysis of Cellular Component subontology. It shows identified categories, and node size in range 25-125 interactions.

Figure S3. Network visualization from the 2,717 proteins in a computational representation through KEGG database with enrichment analysis of the metabolic pathways, biological system, cell, organism, or ecosystem, based on genes and genomes. It shows identified categories, and node size in 50-100 interactions.

Figure S4. Enrich profiler plot. The number of the 412 proteins related to each enrichment analysis for each source, Gene Ontology (Biological Process, Cellular Component and Molecular Function), Reactome and KEGG databases. The X-axis shows number of different enriched processes according to each database, the Y-axis plots the significance value obtained in ascending order (-log10 adj-P-value). A dashed cut-off at the top of the graph divides the most significant enriched cellular processes, a black circle with the number of different processes of interest are highlighted according to each database. The list of the representative processes marked arranged by significance value, labeled by ID (Number in the general list of the process), Resource (Database with which the process was enriched), term ID (Identification of the process according to each platform), Term name (name of the enriched process according to each database) and adj-P-value (significance value of the process) shown with number and color scale and arranged in a descending order.

Figure S5. Network comparison via enrichment analysis through Gene Ontology Biological Process source, from up regulated and down regulated proteins from C- versus 5LANM (S5A), 5RINM (S5B), 10LANM (S5C), 10RINM (S5D), and MTX (S5E). Shows term name, biological process Category, fold change bar and node size interactions scale.

Figure S6. Network comparison via enrichment analysis through Gene Ontology Cellular Component source, from up regulated and down regulated proteins from C- versus 5LANM (S6A), 5RINM (S6B), 10LANM (S6C), 10RINM (S6D), and MTX (S6E). Shows term name, Cellular Component Category, fold change bar and node size interactions scale.

Figure S7. Network comparison via enrichment analysis through KEGG database, from up regulated and down regulated proteins from C- versus 5LANM (S7A), 5RINM (S7B), 10LANM (S7C), 10RINM (S7D), and MTX (S7E). Shows term name related to UNIPROT nomenclature, Kyoto Encyclopedia of Genes and Genomes Category, fold change bar and node size interactions scale.

Figure S8. Show network comparison of down regulated (S8A) and up regulated (S8B) proteins in common between 5LANM, 10LANM and, MTX versus negative control.

Figure S9. Protein relationship in common between 5RINM, 10RINM, and MTX versus negative control. Venn diagram (S9A) illustrating overlapping circles: blue (5RINM), yellow (10RINM) and green (MTX); And network comparison (S9B) indicating up regulated or down regulated of the proteins in common or unique processes.

Figure S10. Show network comparison of down regulated (S10A) and up regulated (S10B) proteins in common between 5RINM, 10RINM and, MTX versus negative control.

Figure S11. Network comparison via enrichment analysis through KEGG, show biological process shared or specific of dysregulated proteins from C- versus 5LANM, 5RINM, 10LANM, 10RINM, and MTX. Shows cluster color comparison, term name, biological process, and circle scale based on the number of genes.

Figure S12. Network comparison via enrichment analysis through Gene Ontology, show biological process and up regulated or down regulated proteins shared and specific of dysregulated proteins from C- versus 5LANM, 5RINM, 10LANM, 10RINM, and MTX. Shows cluster color comparison, term name, biological process, and circle scale based on the number of genes.

Figure S13. Network comparison via enrichment analysis through KEGG, show biological process and up regulated or down regulated proteins shared and specific of dysregulated proteins from C- versus 5LANM, 5RINM, 10LANM, 10RINM, and MTX. Shows cluster color comparison, term name, biological process, and circle scale based on the number of genes.

Table S1. Show intersections of the 2717 identified proteins, related to each enrichment analysis for each source, GO (MF, BP, and CC), KEGG and Reactome databases. Show Source, Term Name, Term ID, highlighted, negative log10 adjusted p value, term size, query size, intersection size, effective domain size and intersections.

Table S2. Show name list of all proteins identified (2717 UNIQ ID, UNIPROT ID, Excel tab) and compared of up/down regulated proteins (412 UNIQ ID, UNIPROT-ID, Excel tab) related to UNIQ ID (Gene name), UNIPROT nomenclature, Entrez ID and Protein names.

Table S3. Show 284 proteins identified from comparison between 5LANM, 10LANM and, MTX versus negative control. Exclusively identified down regulated and up regulated for 5LANM, 10LANM and MTX. Common proteins identified down regulated and up regulated for 5LANM & 10LANM; 5LANM & MTX; 10LANM & MTX; and 5LANM, 10LANM & MTX.

Table S4. Show 374 proteins identified from comparison between 5RINM, 10RINM and, MTX versus negative control. Exclusively identified down regulated and up regulated for 5RINM, 10RINM and MTX. Common proteins identified down regulated and up regulated for 5RINM & 10RINM; 5RINM & MTX; 10RINM & MTX; and 5RINM, 10RINM & MTX.

Table S5. Show down regulated or up regulated proteins identified with contrary behavior among treatments between 5RINM, 10RINM and, MTX versus negative control.

Table S6. Show down regulated and up regulated proteins exclusively identified for treatment 5LANM, 10LANM, 5RINM, 10RINM and, MTX versus negative control.
