# Supplementary material for: Quantitative Proteomics and Molecular Mechanisms of Non-Hodgkin Lymphoma Mice Treated with Incomptine A, Part II
Source: Pharmaceuticals (Basel). 2025 Feb 11;18(2):242. doi: 10.3390/ph18020242 (PMC11858899; doi:10.3390/ph18020242)
Supplement: Supplementary file 1 [file pharmaceuticals-18-00242-s001.zip › Table S3.pdf]

| 284 Total identified Proteins | | | | | |
|---|---|---|---|---|---|
| **Exclusively** | **Exclusively** | **# Down** | **Down Proteins name** | **# Up** | **Up Proteins name** |
| **5LANM** | **27 (9.51%)** | **5** | Pdlim3, Hck, Tpm3, Uqcrh, Nt5c3a | **22** | Sh2d1a, S100a4, Cbr2, H2afv, Cd48, Ces1c, Apex1, S100a9, Tfam, Hist1h1e, Hist1h1a, Hist1h1d, Rps15, Sparcl1, Nup62, Taf9b, Prcp, Nup35, Plbd1, Chmp3, Oxct1, Atp6v1f |
| **10LANM** | **45 (15.85%)** | **6** | Tgfb1, Hspb1, Mprip, Lama2, Zmat2, Tsc22d4, | **39** | Cpa3, Mcpt4, Mug1, Oat, Hmgcl, Anxa5, Impdh1, Gla, Rrp1, Atp6v1d, Psme3, Rps23, Phb, Apoa1, Abracl, Alcam, Postn, Zyx, Cd34, Atp1a3, Gng5, Nrd1, Usp47, Map1s, Ccdc90b, Tprkb, Nupl1, Snrpb2, Rpl15, Prpsap1, Gid8, Med8, Ccdc91, Agk, Prelp, Hmgn5, Htra1, Lypla2, Ap3b1 |
| **MTX** | **84 (29.58%)** | **46** | Zc3h13, Ddost, Gbas, Mtco2, Hbb-b2, Aldoa, Ldha, Ptprc, Hsp90ab1, Cd5, Rpl7, Anxa6, Rplp0, Ass1, Cox4i1, Rps2, Rpl18, Slc25a5, Arpc4, Actb, Sec61a1, Rps18, Rps4x, Anxa4, Atp5a1, Vdac2, Ndufa4, Camk2d, Rps9, Ndufa12, Myoz3, Rnpep, Slc25a3, Aco2, Cox6c, Uqcrq, Atp5f1, Rpl11, Spcs2, Rars, Cyc1, Rpl37, Eef1g, Ndufa9, Ndufs7, Vps35 | **38** | Agrn, Kng1, Ig heavy chain V region 441, Mup3, Ig heavy chain V region AC38 205.12, Ttr, Serpina3k, Alad, Gc, Ahsg, Dbi, Stfa3, Rab21, Dynlt3, Tmsb4x, Mtpn, Ppp1cc, Rbp4, Nufip2, Cp, Bst1, Azgp1, Gpalpp1, Gapvd1, F12, Cpped1, Lyve1, Lonp1, Hexim1, Spon1, Hpx, Glo1, Gnpda2, Chmp5, Cpsf1, Hebp1, Myo1c, Eif4h |
| **Shared** | **Shared** | **# Down** | **Down Proteins name** | **# Up** | **Up Proteins name** |
| **5LANM & 10LANM** | **38 (13.38%)** | **9** | Stat1, Mcm4, Acyp2, Isg15, Nars, Pdlim5, Ing1, Iigp1, Fxr2 | **29** | Rpl21, Hax1, Akap2, Sp3, H3f3a, Itgam, Serpina1a, Ltf, Apoe, HIST1H2BL, Apcs, Hist1h1c, H2afx, Mt3, Apod, Hist1h4a, Csrp1, Casp7, Babam1, Polr2e, Nup54, Pgam5, Hist1h2af, Commd10, Arpin, Prorsd1, Pycrl, H2afy, Mecp2 |
| **5LANM& MTX** | **15 (5.28%)** | **2** | Vdac1, UPF0609 | **13** | Tcn2, Afm, Cfd, Ig gamma-3 chain C region, Alb, Mup2, Serpina1d, Cd55, Cd300lh, Tlk1, Rbm34, Tf, Fetub |
| **10LANM & MTX** | **10 (3.52.%)** | **3** | Rpl10, Elmo1, Fhl3 | **7** | Ig kappa chain V-V region HP 93G7, Apoa4, Cma1, Serpina1b, Cfi, Il1rap, Bcl7a |
| **5LANM, 10LANM, & MTX** | **65 (22.89%)** | **60** | Myom3, Ttn, Casq1, Pgam2, Actn3, Mb, Slc4a1, Myl1, Ckm, Myl3, Fabp3, Tnni2, Myh3, Myh8, Rel, Eno2, Tnnc2, Eno3, Cryab, Pvalb, Acsl1, Cfl2, Slc25a4, Myl2, Tpm1, Tpm2, Acta1, Fhl1, Mylpf, Rac2, Pdlim7, Myh4, Myh1, Mybpc2, Myom1, Ifit3, Ckmt2, Srl, Ifi44, Spg20, Atp2a1, Flnc, Myh7, Dhrs1, Ubqln4, Ndufb4, Mrps22, Chchd2, Ccdc101, Zfyve19, Actn2, Myot, Myoz1, Ldb3, Tmod4, Tnnt3, Ak1, Pygm, Sh3bgr, Sucla2 | **5** | Marcks, Rplp1, Phactr4, Timd4, Gar1 |
