# Supplementary material for: Quantitative Proteomics and Molecular Mechanisms of Non-Hodgkin Lymphoma Mice Treated with Incomptine A, Part II
Source: Pharmaceuticals (Basel). 2025 Feb 11;18(2):242. doi: 10.3390/ph18020242 (PMC11858899; doi:10.3390/ph18020242)
Supplement: Supplementary file 1 [file pharmaceuticals-18-00242-s001.zip › Table S4.pdf]

374 Total identified Proteins					
**Exclusively**	**Exclusively**	**# Down**	**Down Proteins name**	**# Up**	**Up Proteins name**
**5RINM**	**61 (16.31%)**	**24**	Rnf213, Api5, Hbb-b1, Hck, Cox5a, Hspb1, Tpm3, Stat1, Stt3a, Tuba4a, Uqcrh, Uba1, C17orf62, Tpm4, Mybbp1a, Ipo5, Nars, Tmx3, Tmx1, Lrrc59, Nampt, Aldh6a1, Clic4, Iigp1	**37**	Krt10, Purb, Akap2, Smarce1, Rnf13, Crym, Pdlim1, Wdr1, Ig epsilon chain C region, Plg, Mcpt4, Il6ra, Snrpb, Cirbp, Snrpe, Snrpd2, Snrpd3, Casp3, Ckb, Babam1, Zyx, Nup62, Taf9b, Atp1a3, Slc7a6os, Srek1, Iws1, C19orf66, Nup35, Pdxdc1, Nup37, Hnrnpa0, Kynu, Mrto4, Pycrl, Nudt5, Ruvbl2
**10RINM**	**117 (31.28%)**	**17**	Pdlim3, Tgfb1, Eci1, Vcp, Acyp2, Mprip, Fbln1, Cmpk2, Hp, Cmbl, Clybl, Tbcc, Ptgr2, Ptgr1, Nipsnap3b, Tsc22d4, Prosc	**100**	Mocs3, Clta, Gfra2, Rpl21, Hax1, Adh1, Ig heavy chain V-III region T957, Cbr2, Hmgn2, H2afv, HIST1H2BL, H1f0, Lamp1, Apcs, Rpl7a, S100a6, Cpa3, Hist1h1c, Hmga1, Rpl13a, Mt3, Apex1, Fbl, Tfam, Rpl28, Hist1h1e, Hist1h1a, Hist1h1b, Hist1h1d, Gpx3, Rpl6, Rpl29, Rpl13, Rpl36, Cav1, Apod, Ctsw, Cyb5a, Atp6v1d, Sf3b6, Rpl26, Rpl27, Rpl36a, Rps8, Rps13, Rps15, Rps25, Rpl31, Rpl32, Rpl8, Sparcl1, Rpl19, Casp7, Rplp2, Apoa1, Col6a2, Nucb1, Top1, Col6a1, Hp1bp3, Marco, Alcam, Cd34, Cltb, Rpl35, Gng5, Rpl24, Arrb1, Pgam5, Ccdc90b, Rbm4b, Plin1, Hist1h2af, Cd209b, Commd10, Manba, Bloc1s4, Basp1, Marc2, Rpl17, Atg3, UPF0568, Mrpl11, Snrpb2, Rpl14, Chtop, Rps19, Prpsap1, Atp6v1f, Rpl34, Gid8, Prorsd1, Rp2, Eny2, Rpl38, Hmgn5, Hdgfrp3, Zbp1, H2afy, Baz1b
**MTX**	**54 (14.44%)**	**18**	Gbas, Ptprc, Cd5, Anxa6, Rplp0, Ass1, Rps2, Arpc4, Rps18, Rps4x, Atp5a1, Ndufa4, Camk2d, Aco2, Cox6c, Uqcrq, Rpl11, Ndufs7	**36**	Agrn, Tcn2, Ig heavy chain V region 441, Ig gamma-3 chain C region, Mup3, Ig heavy chain V region AC38 205.12, Ttr, Serpina3k, Gc, Cma1, Ahsg, Dbi, Stfa3, Rab21, Tmsb4x, Mtpn, Ppp1cc, Serpina1d, Nufip2, Azgp1, Gpalpp1, Gapvd1, F12, Cpped1, Lyve1, Rbm34, Lonp1, Hexim1, Spon1, Hpx, Glo1, Bcl7a, Cpsf1, Hebp1, Myo1c, Eif4h
**Shared**	**Shared**	**# Down**	**Down Proteins name**	**# Up**	**Up Proteins name**
**5RINM & 10RINM**	**22 (5.88%)**	**6**	Mcm4, Isg15, Pdlim5, Nt5c3a, Ing1, Fxr2	**16**	Sp3, H3f3a, Cfh, S100a4, Alpl, Cd48, H2afx, Hist1h4a, Serping1, Csrp1, Orm1, Polr2e, Nup54, Ssbp1, Arpin, Mecp2
**5RINM& MTX**	**44 (11.76%)**	**30**	Zc3h13, Ddost, Mtco2, Hbb-b2, Ldha, Hsp90ab1, Cox4i1, Acsl1, Slc25a5, Actb, Sec61a1, Rac2, Vdac2, Vdac1, Rps9, Rpl10, Ndufa12, Elmo1, UPF0609, Myoz3, Slc25a3, Dhrs1, Atp5f1, Spcs2, Rars, Cyc1, Eef1g, Ndufa9, Vps35, Sucla2	**14**	Kng1, Afm, Alb, Alad, Mup2, Serpina1b, Dynlt3, Rbp4, Cfi, Bst1, Tlk1, Gnpda2, Gar1, Chmp5
**10RINM & MTX**	**14 (3.74.%)**	**4**	Aldoa, Anxa4, Rnpep, Fhl3, (plus Rpl7, Rpl37)	**8**	Ig kappa chain V-V region HP 93G7, Apoa4, Rplp1, Cp, Cd55, Il1rap, Cd300lh, Timd4, (plus Rpl7, Rpl37)
**5RINM, 10RINM, & MTX**	**62 (16.58%)**	**56**	Myom3, Ttn, Casq1, Pgam2, Actn3, Mb, Slc4a1, Myl1, Ckm, Myl3, Fabp3, Tnni2, Myh3, Myh8, Rel, Eno2, Tnnc2, Eno3, Cryab, Pvalb, Cfl2, Slc25a4, Myl2, Tpm1, Tpm2, Acta1, Fhl1, Mylpf, Pdlim7, Myh4, Myh1, Mybpc2, Myom1, Ifit3, Ckmt2, Srl, Ifi44, Spg20, Atp2a1, Flnc, Myh7, Ubqln4, Ndufb4, Mrps22, Chchd2, Ccdc101, Zfyve19, Actn2, Myot, Myoz1, Ldb3, Tmod4, Tnnt3, Ak1, Pygm, Sh3bgr, (plus Rpl18)	**5**	Cfd, Marcks, Phactr4, Tf, Fetub, (plus Rpl18)
