# Supplementary material for: Quantitative Proteomics and Molecular Mechanisms of Non-Hodgkin Lymphoma Mice Treated with Incomptine A, Part II
Source: Pharmaceuticals (Basel). 2025 Feb 11;18(2):242. doi: 10.3390/ph18020242 (PMC11858899; doi:10.3390/ph18020242)
Supplement: Supplementary file 1 [file pharmaceuticals-18-00242-s001.zip › Table S5.pdf]

Contrary behavior proteins	# Down	Down Proteins name	# Up	Up Proteins name
**5RINM**	**1**	Rpl18		
**10RINM**			**3**	Rpl7, Rpl18, Rpl37
**MTX**	**3**	Rpl7, Rpl18, Rpl37		
