# Supplementary material for: Quantitative Proteomics and Molecular Mechanisms of Non-Hodgkin Lymphoma Mice Treated with Incomptine A, Part II
Source: Pharmaceuticals (Basel). 2025 Feb 11;18(2):242. doi: 10.3390/ph18020242 (PMC11858899; doi:10.3390/ph18020242)
Supplement: Supplementary file 1 [file pharmaceuticals-18-00242-s001.zip › Table S6.pdf]

Exclusively	Total	# Down	Down Proteins name	# Up	Up Proteins name
5LANM	7	0		7	Sh2d1a, Ces1c, S100a9, Prcp, Plbd1, Chmp3, Oxct1
10LANM	29	2	Lama2, Zmat2	27	Mug1, Oat, Hmgcl, Anxa5, Impdh1, Gla, Rrp1, Psme3, Rps23, Phb, Abracl, Postn, Nrd1, Usp47, Map1s, Tprkb, Nupl1, Rpl15, Med8, Ccdc91, Agk, Prelp, Htra1, Lypla2, Ap3b1
5RINM	45	17	Rnf213, Api5, Hbb-b1, Cox5a, Stt3a, Tuba4a, Uba1, C17orf62, Tpm4, Mybbp1a, Ipo5, Tmx3, Tmx1, Lrrc59, Nampt, Aldh6a1, Clic4	28	Krt10, Purb, Smarce1, Rnf13, Crym, Pdlim1, Wdr1, Ig epsilon chain C region, Plg, Il6ra, Snrpb, Cirbp, Snrpe, Snrpd2, Snrpd3, Casp3, Ckb, Slc7a6os, Srek1, Iws1, UPF0515, Pdxdc1, Nup37, Hnrnpa0, Kynu, Mrto4, Nudt5, Ruvbl2
10RINM	78	12	Eci1, Vcp;VCP, Fbln1, Cmpk2, Hp, Cmbl, Clybl, Tbcc, Ptgr2, Ptgr1, Nipsnap3b, Prosc	66	Mocs3, Clta, Gfra2, Adh1, HVM31, Hmgn2, H1f0, Lamp1, Rpl7a, S100a6, Hmga1, Rpl13a, Fbl, Rpl28, Hist1h1b, Gpx3, Rpl6, Rpl29, Rpl13, Rpl36, Cav1, Ctsw, Cyb5a, Sf3b6, Rpl26, Rpl27, Rpl36a, Rps8, Rps13, Rps25, Rpl31, Rpl32, Rpl8, Rpl19, Rplp2, Col6a2, Nucb1, Top1, Col6a1, Hp1bp3, Marco, Cltb, Rpl35, Rpl24, Arrb1, Rbm4b, Plin1, Cd209b, Manba, Bloc1s4, Basp1, Marc2, Rpl17, Atg3, UPF0568, Mrpl11, Rpl14, Chtop, Rps19, Rpl34, Rp2, Eny2, Rpl38, Hdgfrp3, Zbp1, Baz1b
MTX	48	18	Gbas, Ptprc, Cd5, Anxa6, Rplp0, Ass1, Rps2, Arpc4, Rps18, Rps4x, Atp5a1, Ndufa4, Camk2d, Aco2, Cox6c, Uqcrq, Rpl11, Ndufs7	30	Agrn, HVM36, Mup3, I HVM51, Ttr, Serpina3k, Gc, Ahsg, Dbi, Stfa3, Rab21, Tmsb4x, Mtpn, Ppp1cc, Nufip2, Azgp1, Gpalpp1, Gapvd1, F12, Cpped1, Lyve1, Lonp1, Hexim1. Spon1. Hpx, Glo1, Cpsf1, Hebp1, Myo1c, Eif4h
