# Supplementary figures and images for: Quantitative Proteomics and Molecular Mechanisms of Non-Hodgkin Lymphoma Mice Treated with Incomptine A, Part II
Source: Pharmaceuticals (Basel). 2025 Feb 11;18(2):242. doi: 10.3390/ph18020242 (PMC11858899; doi:10.3390/ph18020242)

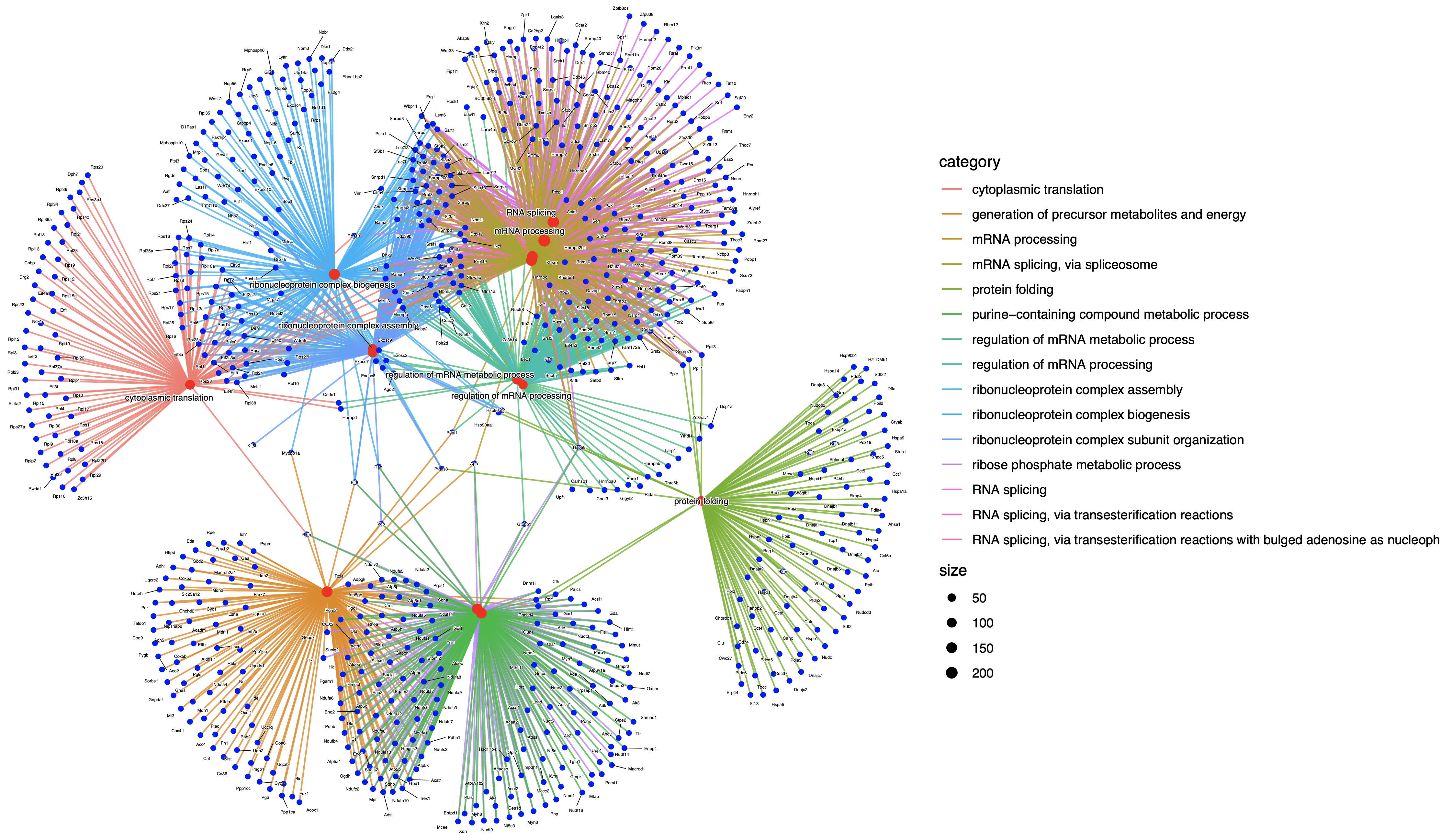

Supplement: Supplementary file 1 [file pharmaceuticals-18-00242-s001.zip › Figure S1.png]

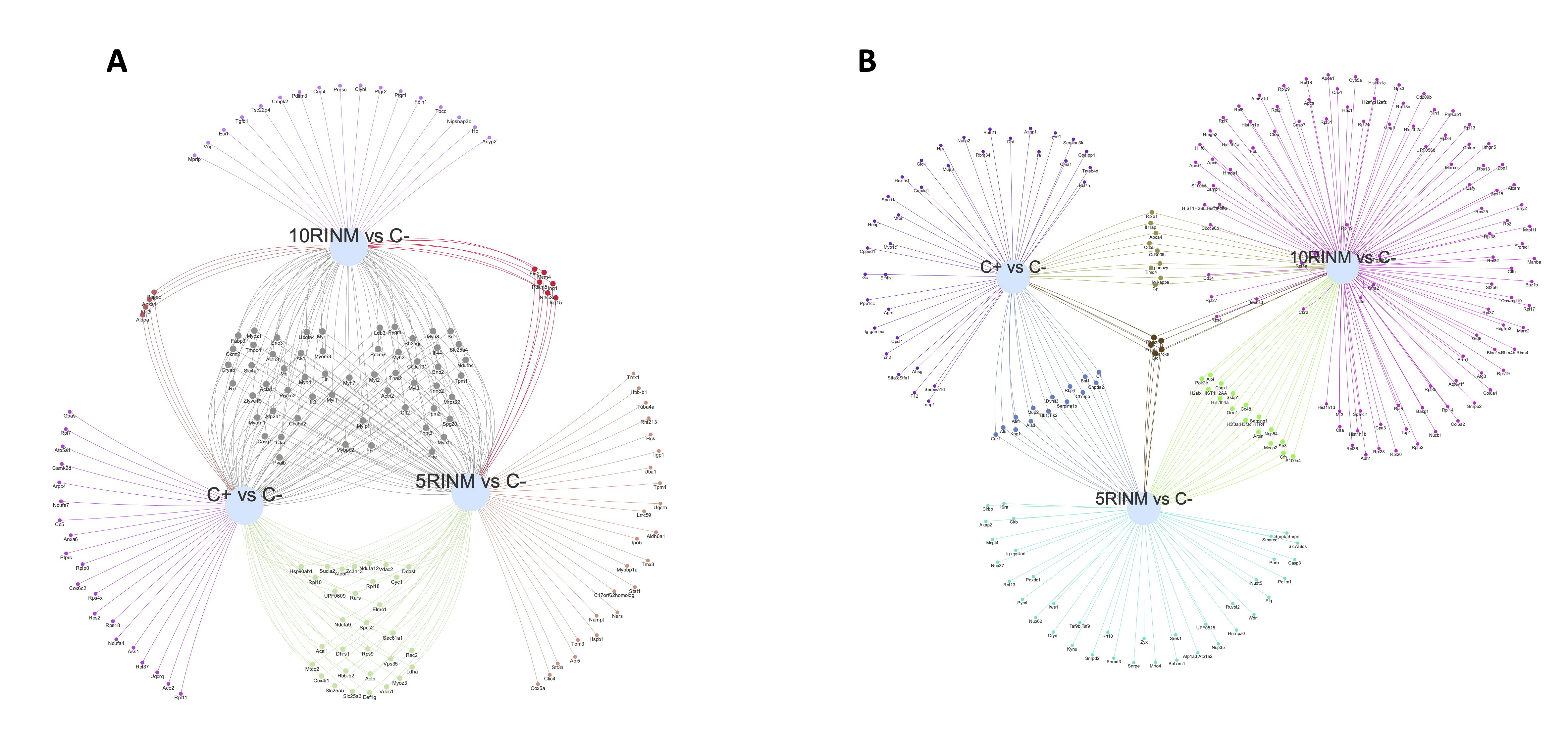

Supplement: Supplementary file 1 [file pharmaceuticals-18-00242-s001.zip › Figure S10.png]

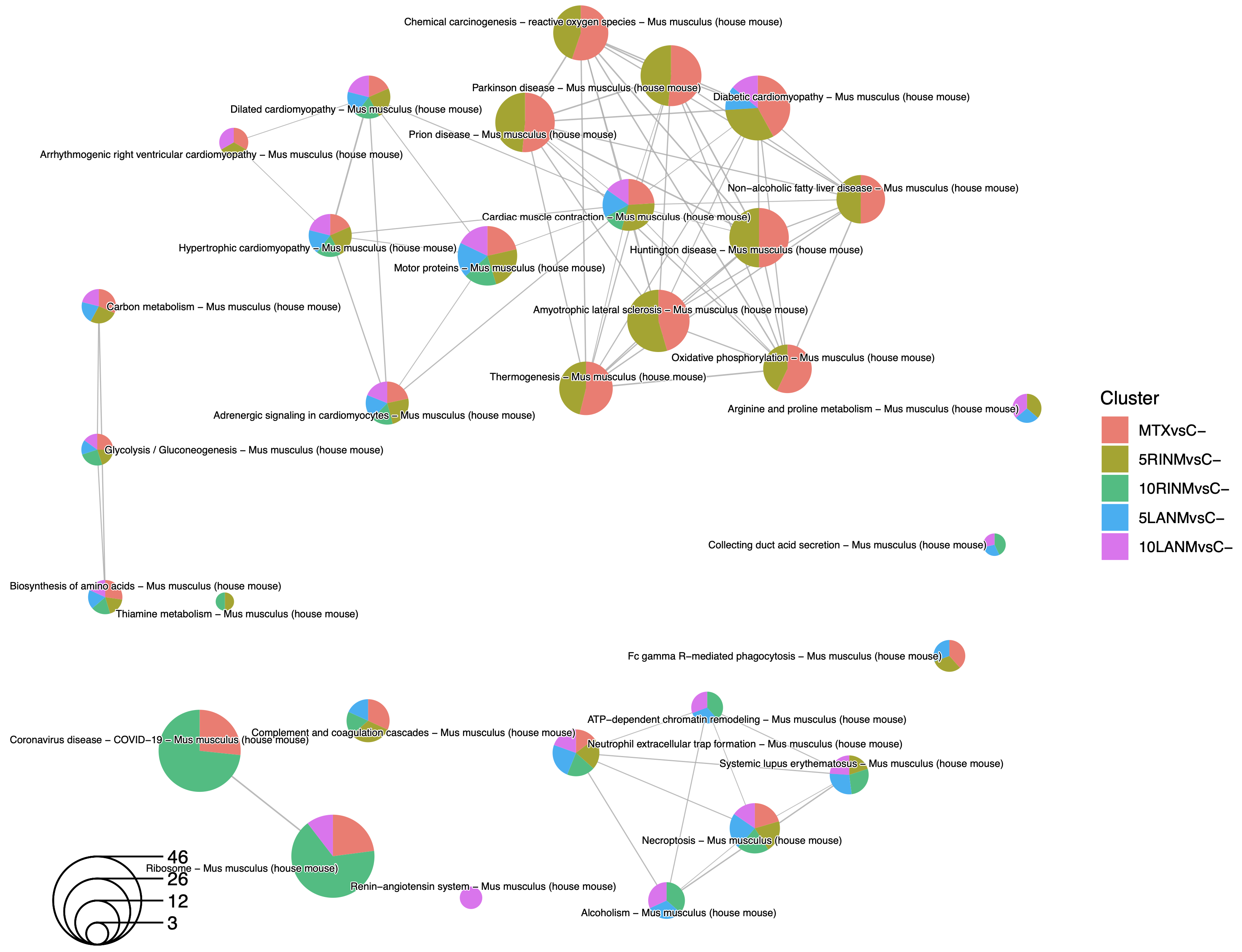

Supplement: Supplementary file 1 [file pharmaceuticals-18-00242-s001.zip › Figure S11.png]

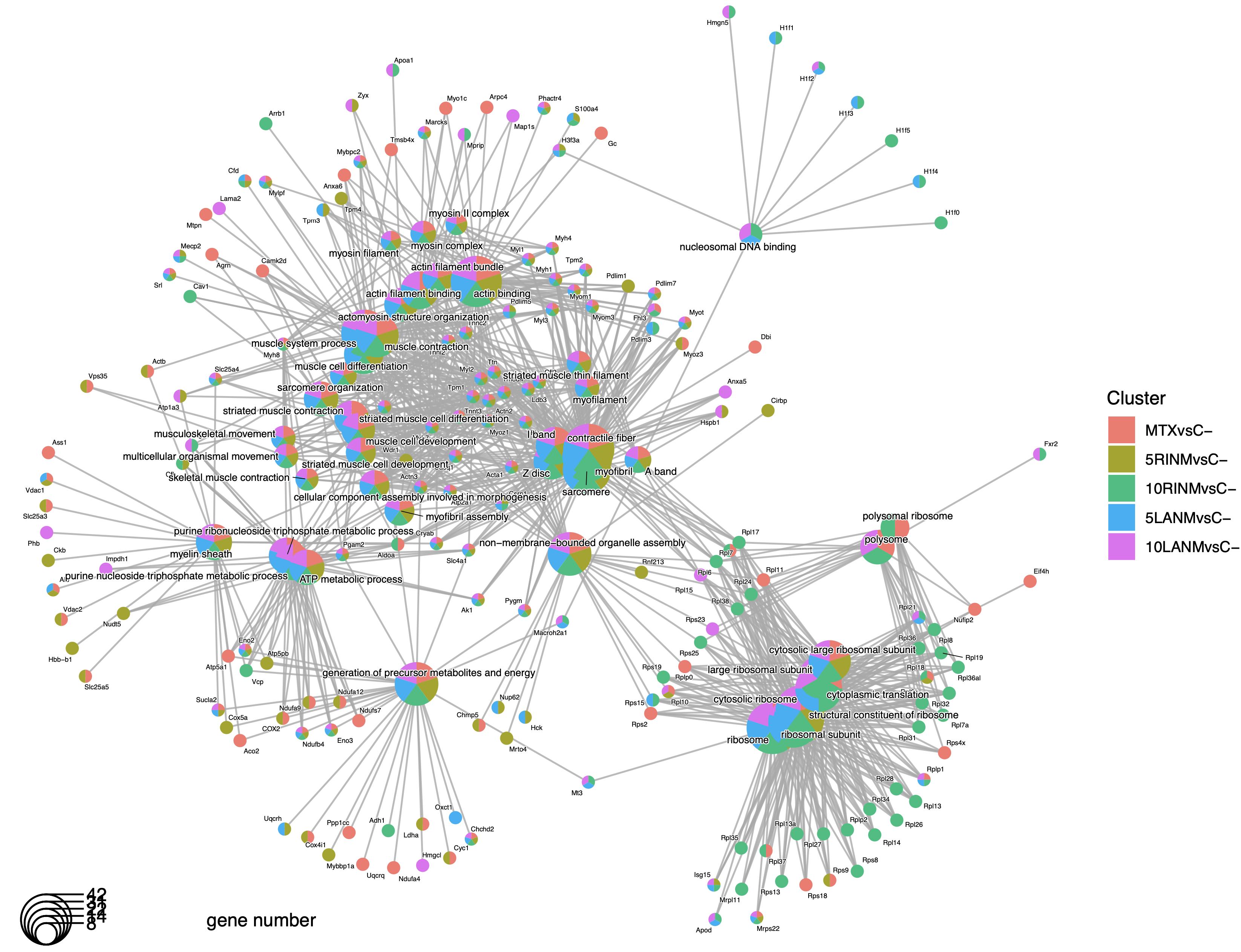

Supplement: Supplementary file 1 [file pharmaceuticals-18-00242-s001.zip › Figure S12.png]

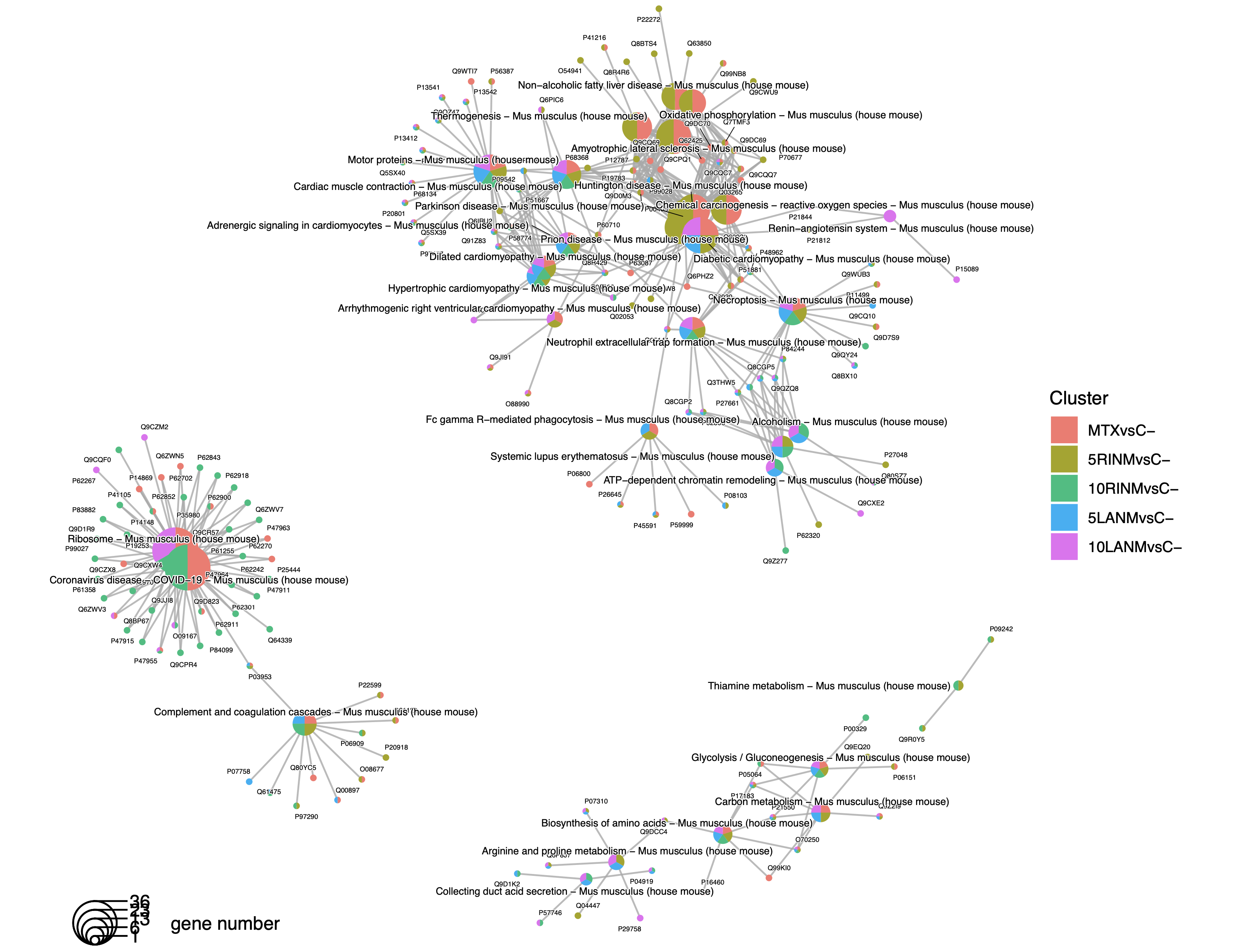

Supplement: Supplementary file 1 [file pharmaceuticals-18-00242-s001.zip › Figure S13.png]

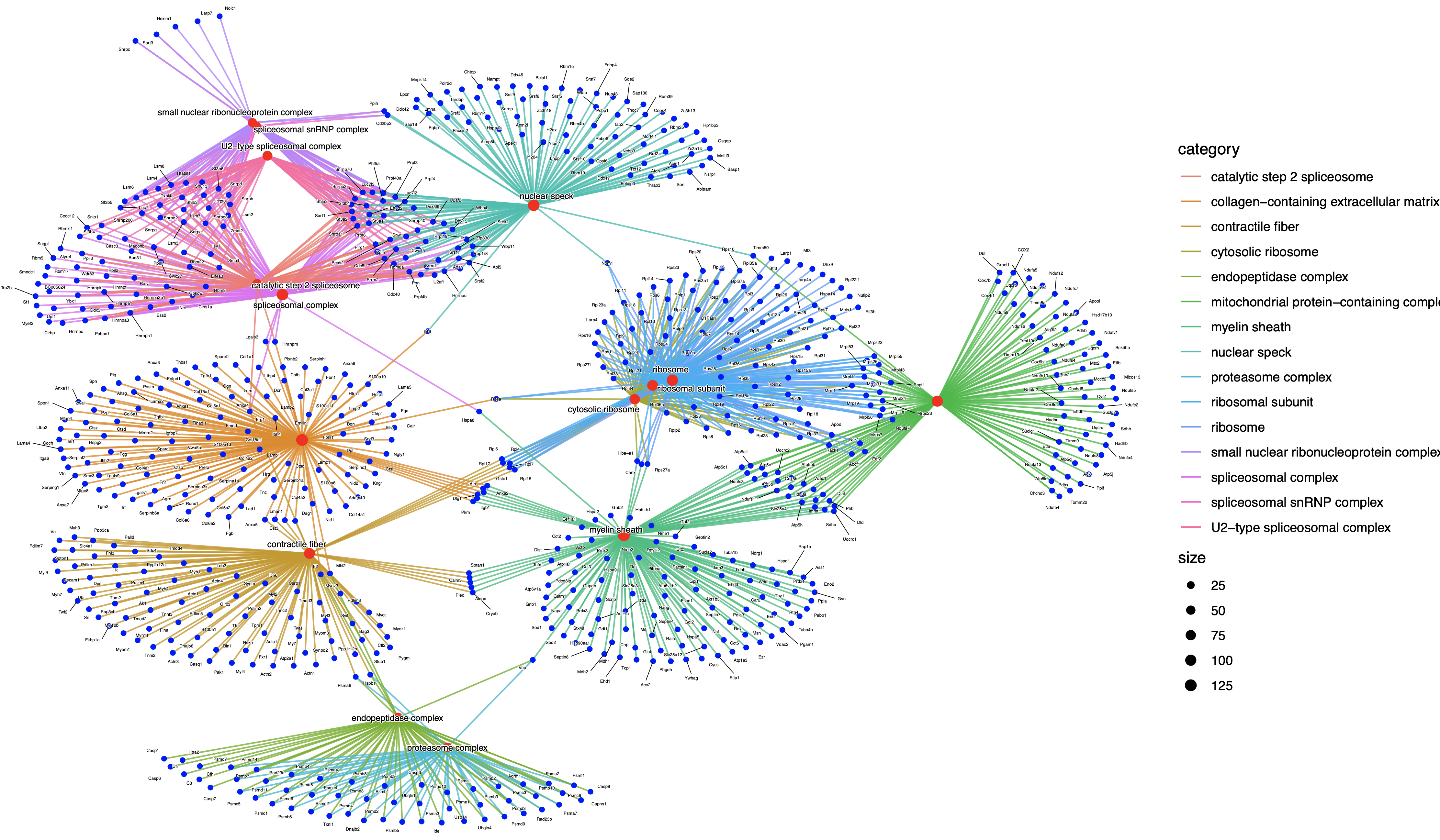

Supplement: Supplementary file 1 [file pharmaceuticals-18-00242-s001.zip › Figure S2.png]

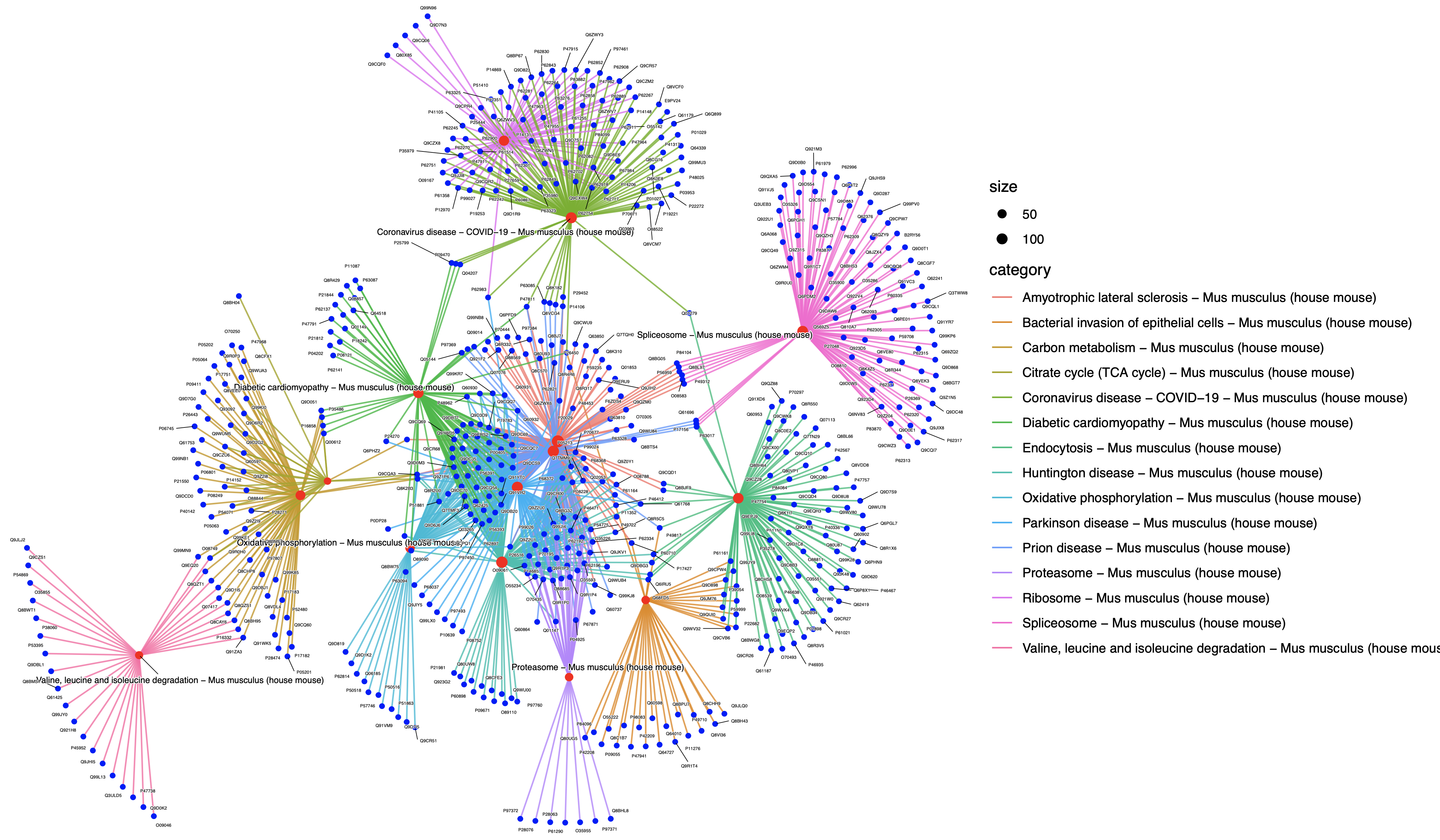

Supplement: Supplementary file 1 [file pharmaceuticals-18-00242-s001.zip › Figure S3.png]

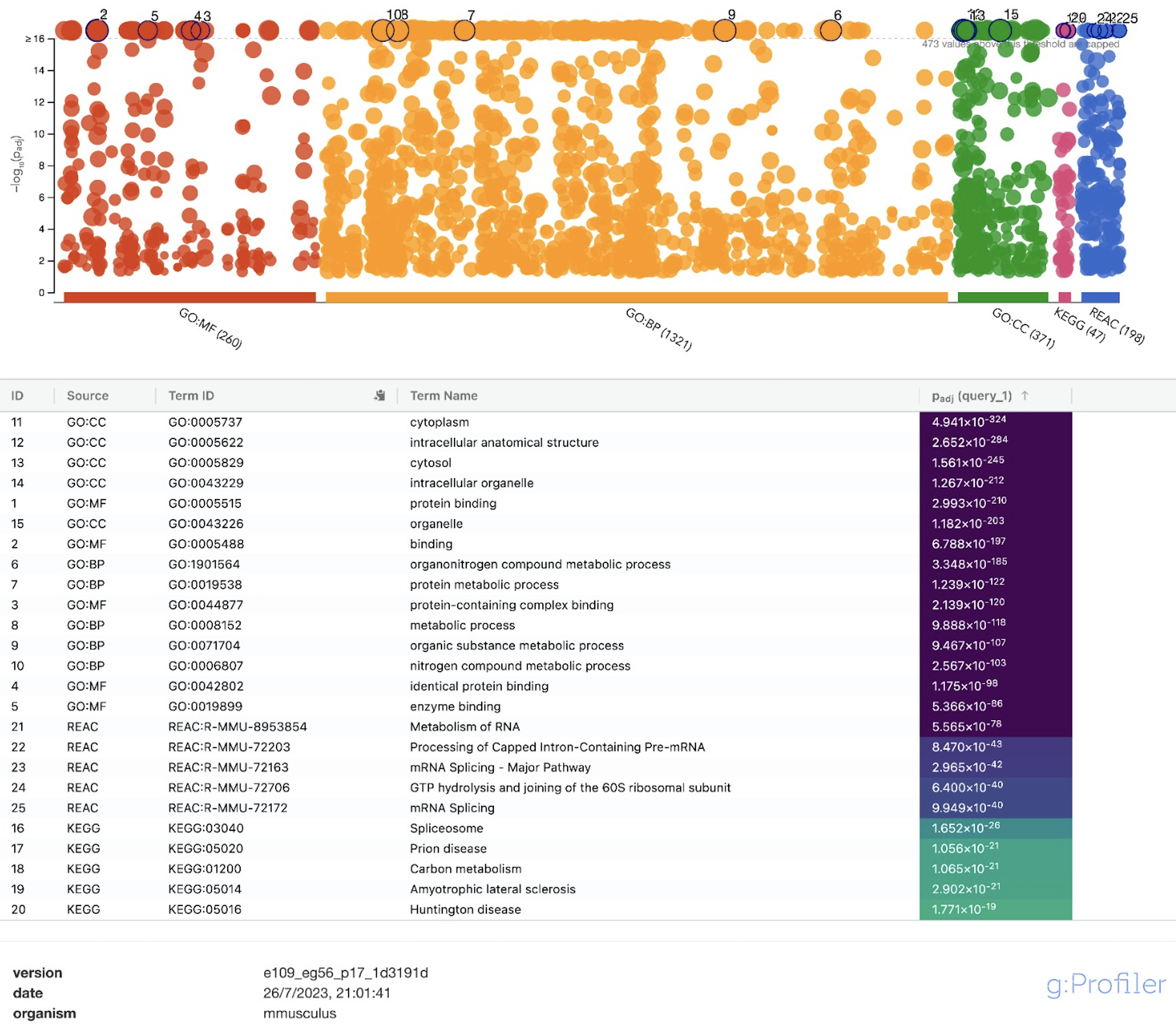

Supplement: Supplementary file 1 [file pharmaceuticals-18-00242-s001.zip › Figure S4.png]

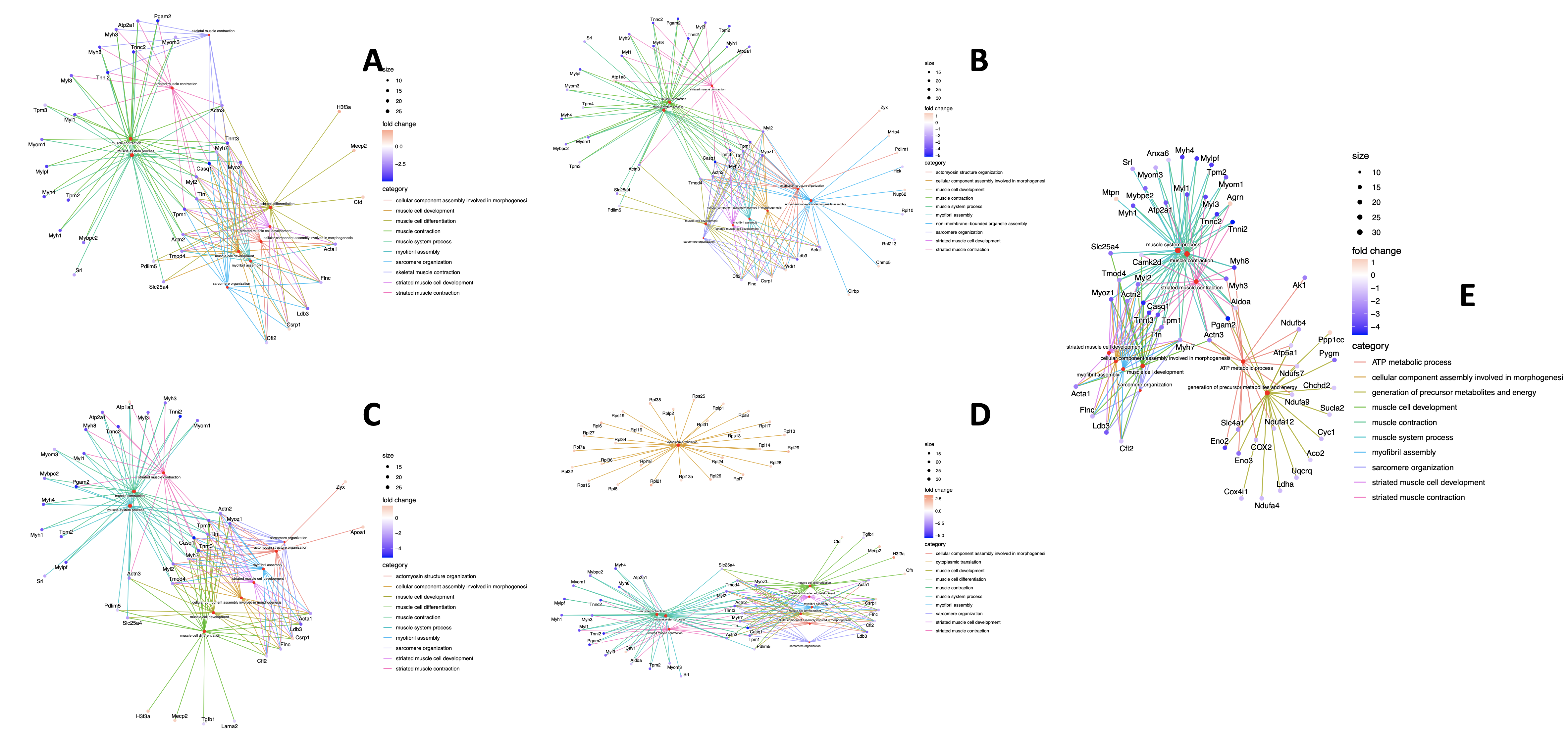

Supplement: Supplementary file 1 [file pharmaceuticals-18-00242-s001.zip › Figure S5.png]

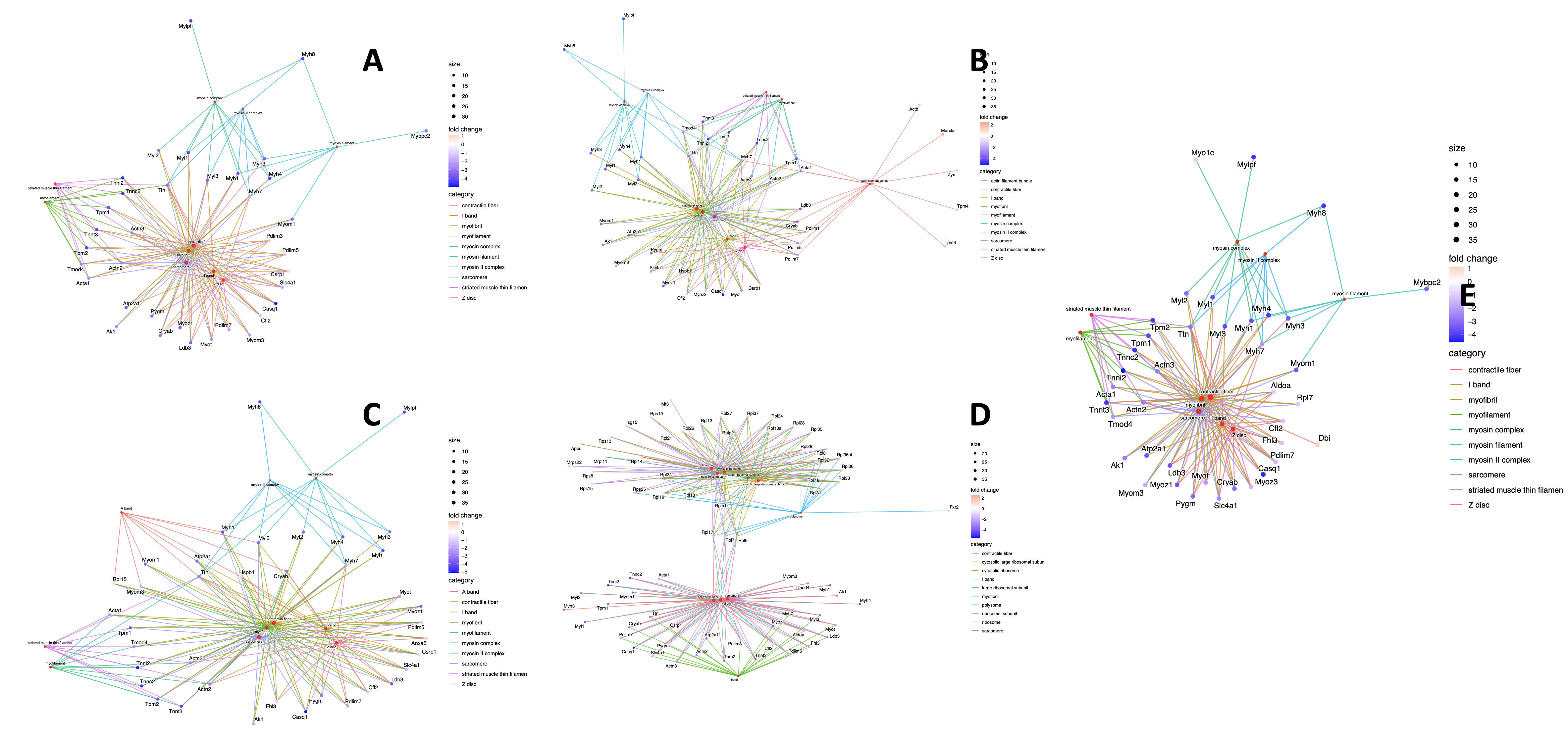

Supplement: Supplementary file 1 [file pharmaceuticals-18-00242-s001.zip › Figure S6.png]

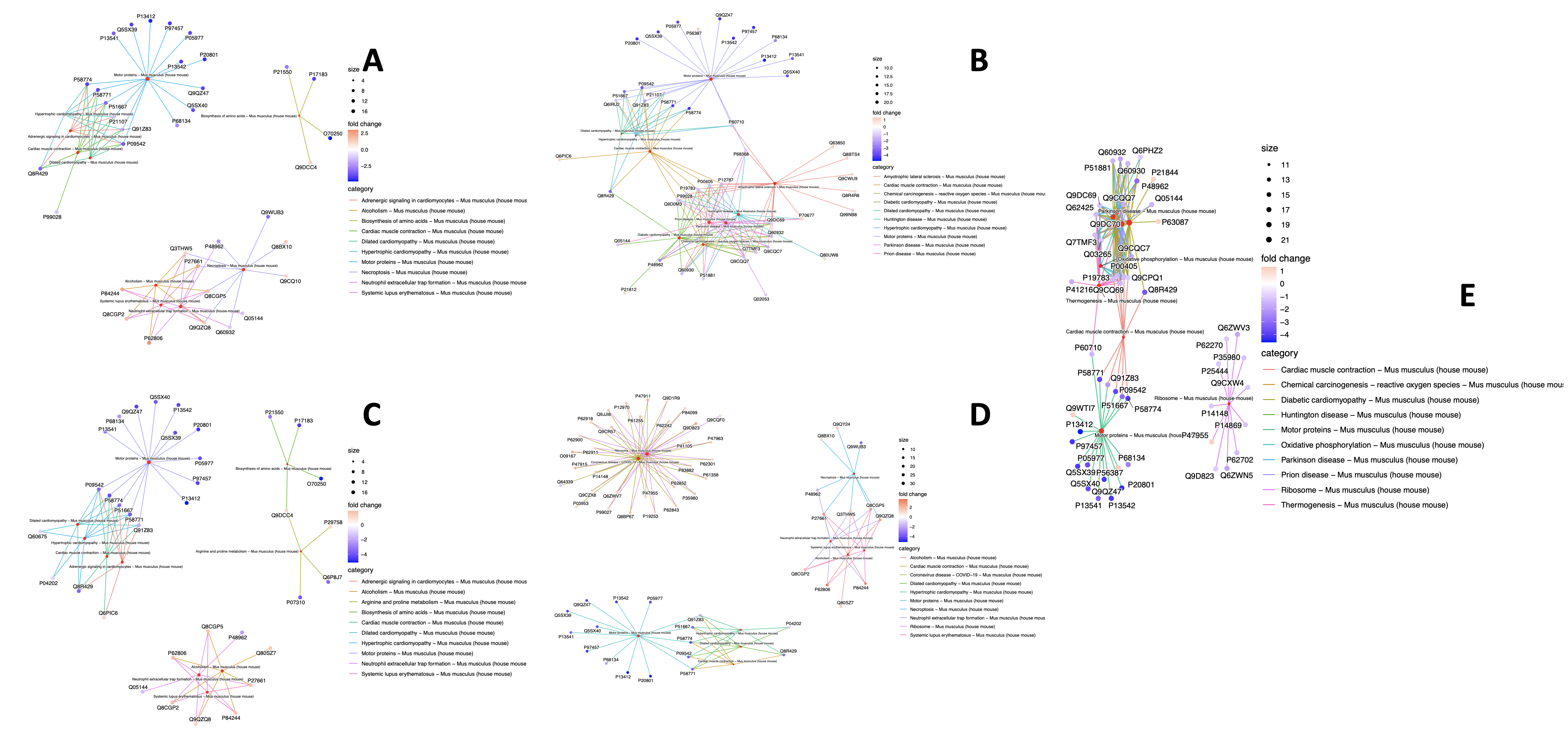

Supplement: Supplementary file 1 [file pharmaceuticals-18-00242-s001.zip › Figure S7.png]

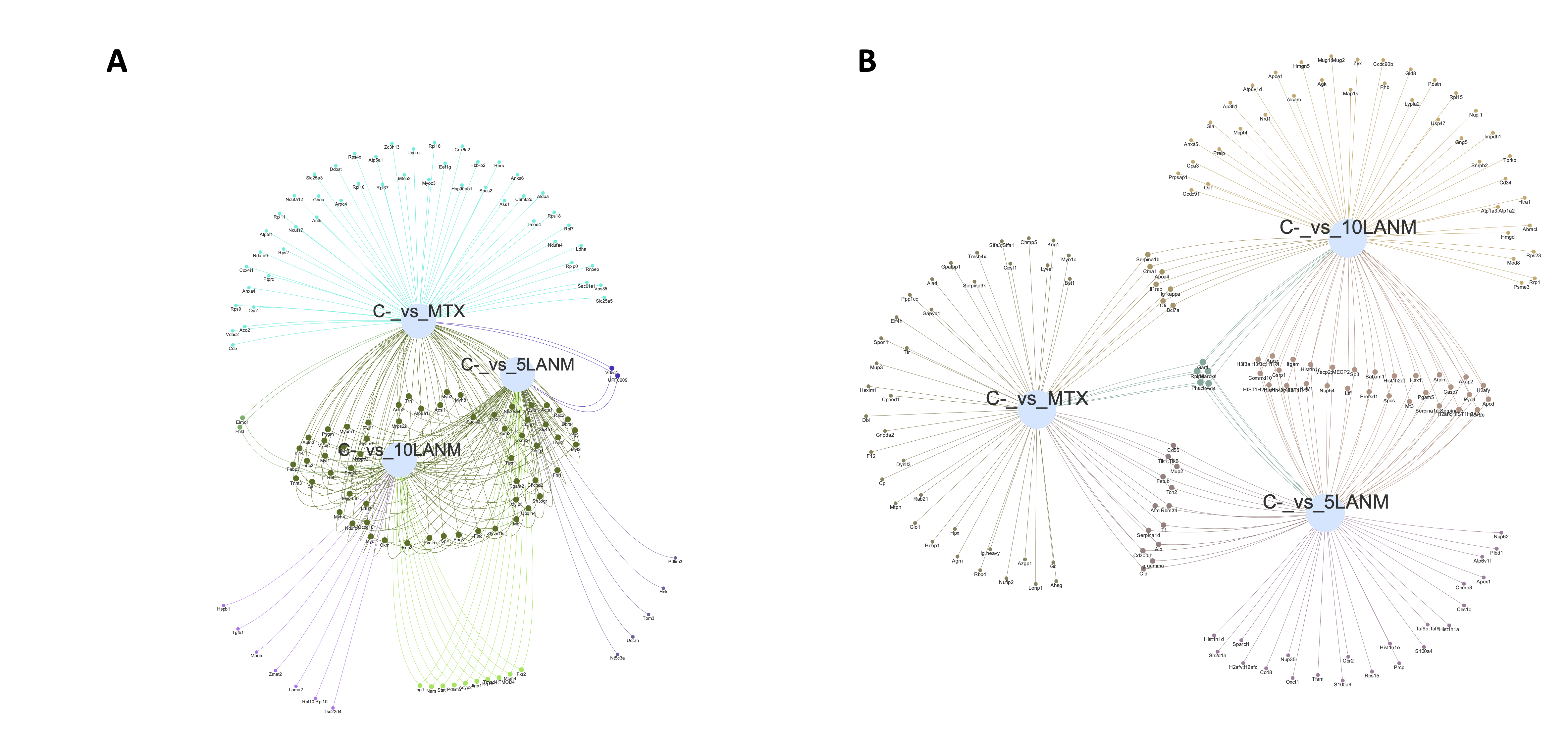

Supplement: Supplementary file 1 [file pharmaceuticals-18-00242-s001.zip › Figure S8.png]

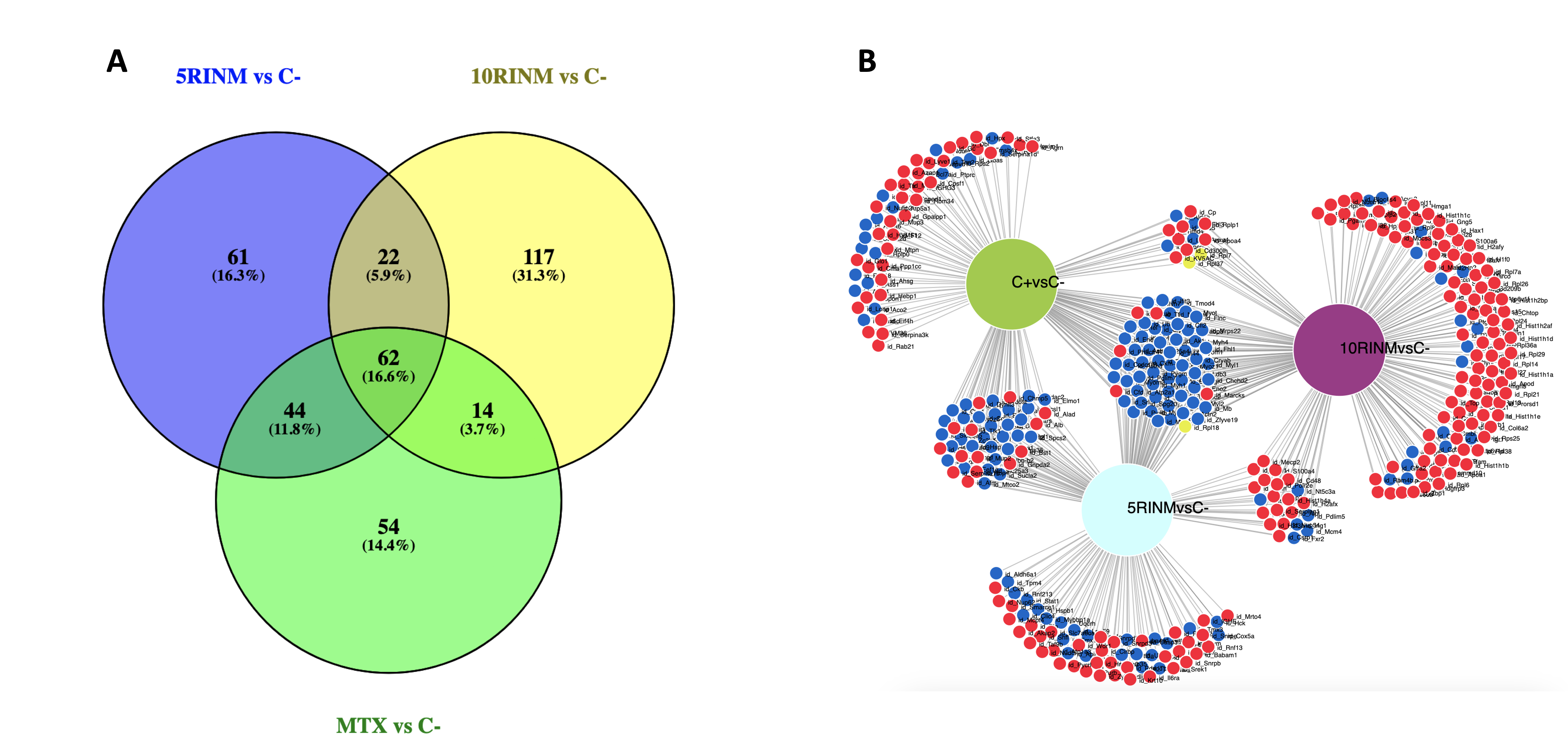

Supplement: Supplementary file 1 [file pharmaceuticals-18-00242-s001.zip › Figure S9.png]
